# RETRACTED ARTICLE: miR-25-3p promotes proliferation and inhibits autophagy of renal cells in polycystic kidney mice by regulating ATG14-Beclin 1

**DOI:** 10.1080/0886022X.2026.2667001

**Published:** 2026-05-06

We, the Editor and Publisher of *Renal Failure* have retracted the following article:

Liu, G., Kang, X., Guo, P., Shang, Y., Du, R., Wang, X., … Kong, F. (2020). miR-25-3p promotes proliferation and inhibits autophagy of renal cells in polycystic kidney mice by regulating ATG14-Beclin 1. *Renal Failure*, *42*(1), 333–342. https://doi.org/10.1080/0886022X.2020.1745236

After publication, concerns were raised by a third party regarding the integrity of the Figures 1 and 2 of the article.

Further investigation conducted by the Journal and Publisher confirmed significant methodological ­concerns that affect the integrity of the Western blot data shown in Figures 1 and 2. Additional inconsistencies between the data shown in Figure 2 and the corresponding figure legend were also identified.

It has also come to our attention that the published version of the article significantly differs from the version that was peer reviewed and accepted. As such the published article was not peer-reviewed appropriately, in line with the Journal’s peer review standards and policy.

When approached for an explanation, the authors did not respond to the queries raised by the Journal and Publisher.

As the stringency of the peer review process is core to the integrity of the publication process, and verifying the validity of published work is core to the integrity of the scholarly record, we are therefore, retracting the article. The corresponding author has been informed.

We have been informed in our decision-making by our editorial policies and the COPE guidelines.

The retracted articles will remain online to maintain the scholarly record, but they will be digitally watermarked on each page as ‘Retracted’.

